# Willingness to join community based health insurance and its determinants in East Gojjam zone, Northwest Ethiopia

**DOI:** 10.1186/s13104-019-4060-3

**Published:** 2019-01-17

**Authors:** Getiye Dejenu Kibret, Cheru Tesema Leshargie, Fasil Wagnew, Animut Alebel

**Affiliations:** College of Health Sciences, Debre Markos University, Debre Markos, Ethiopia

**Keywords:** CBHI, Ethiopia, Willingness

## Abstract

**Objective:**

The main purpose of this research was to determine the magnitude of willingness to join to community based health insurance (CBHI) and to identify factors associated with it.

**Results:**

A total of 604 study participants responded for the interviews, making the response rate 98.2%. All in all, 492 (81.5%) of the study participant households were willing to join the CBHI scheme. Households which had experience of borrowing for medical expenses within the last 12 months prior to the study were 2.7 times more likely to join CBHI scheme than those who didn’t have borrowed (AOR = 2.65; 95% CI 1.03, 6.83). Female headed households were 2.7 times more likely to take up the scheme compare to male headed households (AOR = 2.74; 95% CI 1.18, 6.37). High proportion of households was willing to join the CBHI scheme in the study area. Educational status of household head, experience of borrowing for medical expenses, sex of household head, household animal asset as measured by tropical livestock unit were factors found to be associated with willingness to take up CBHI scheme.

## Introduction

A health insurance scheme refers to pooling of prepaid funds in a way that allows for risks to be shared. The health insurance scheme particularly suitable for the rural poor and the informal sector in low and middle income countries is community-based health insurance (CBHI), that is, insurance schemes operated by organizations other than governments or private for profit companies [1].

In Ethiopia the top leading causes of mortality were easily preventable communicable diseases such as malaria, pneumonia and respiratory tract infections [2]. In the other hand, utilization of modern health care services is limited [3]. One of the main reasons for low utilization of healthcare services is the out of pocket payment [4], where around 38.5% of the total health expenditures were covered through out-of-pocket charges and the remaining costs are covered from the revenue based funds, government subsidies and international funders in the country [3].

The Ethiopian government had already initiated health insurance schemes, social health insurance [5] for the formal sector (yet not implemented until the end of 2015), and community-based health insurance (CBHI) for citizens in the informal and agriculture sectors. The necessary legal frameworks are already in place for the implementation of CBHI schemes as well as for initiation of the SHI program [6]. The announcement ties into the country’s larger health sector financing strategy, which aims to improve financing services for rural populations, better define fee waivers, increase cost recovery and reform the payment system [7].

The Ethiopian CBHI benefits packages include all family health services and curative cares including inpatient, outpatient services and acute illnesses [8].

The CBHI scheme is expected to cover the larger segment (83.6%) of Ethiopian population. The joining of CBHI is based on voluntary decision of the households, which can be affected by different socio-economic and awareness level factors [4]. In the introduction of such a nationwide program, it is crucial to have baseline data regarding the prevailing demands and focus areas for scale up.

In order to achieve the universal health coverage strategy, both the community based and social health insurance schemes were planned to cover about 50% of the population at the end of the 2015 in Ethiopia [9]. But the coverage of CBHI by the end of 2015 was far below the plan and the social health insurance did not still started to implement.

Therefore, it would be important to have estimated magnitude of volunteers to engage in the scheme and what factors are hampering households from joining the CBHI scheme. There is also paucity of studies in Ethiopia regarding the willingness to be enrolled in the scheme.

The objective of this study was therefore, to determine the magnitude of willingness to join and determinant factors in East Gijjam administrative zone, northwest Ethiopia.

## Main text

### Methods

#### Study design and area

Cross sectional study design was implemented. The study was conducted in East Gojam zone of Amhara regional state, Ethiopia from July 27, 2014 to August 30, 2014. East Gojjam zone is one of the 11 zones of Amhara regional state. The zone consists of 18 districts and 4 administrative towns [10].

#### Population

All households in East Gojjam administrative Zone were considered as source population and all household heads in the randomly selected districts were study populations.

#### Sample size and sampling method

All in all, 615 samples were proposed for data source determined using single population proportion formula. Stratified multistage sampling method was used in order to reach to the study participants. The sample was allocated proportionally to each district for both urban and rural areas. Finally, systematic random sampling method was used to access the interviewee.

#### Data collection procedures

Data was collected using structured questionnaires. The questionnaires were adapted from literatures developed for similar purposes by different authors and were reviewed to suit it to the local situation. The questionnaire was prepared first in English and then it was translated to Amharic version. The data were collected by trained fresh BSC graduated health professionals.

To elicit the willingness to join, first data collectors gave detailed description of the meanings and concepts of CBHI for household heads; what it covers, what it benefits and it costs them. Then they asked about their willingness.

#### Outcome of interest

The primary outcome of interest was willingness to join CBHI and the secondary outcome was estimates of factors associated with willingness to join.

#### Eligibility criteria

All selected household heads in the study area were asked for their actual willingness to pay.

Critically ill household heads that were not able to respond appropriately for the interviews were excluded from the study.

#### Data quality control

In order to maintain quality of data, data collectors and supervisors were trained and questionnaire guide was prepared. Pre-test was done on 5% of the total sample size out of the data collection area.

#### Data processing and analysis

Data were cleaned and fed to EpiData version 3.1 and analysis was done by using STATA 13 statistical software. Logit model was fitted to see the association between willingness to join and the explanatory variables. The significance was declared at p-values of < 0.05 within 95% confidence interval.

#### Operational definition

*Willingness to join*: the willingness of household heads to join the proposed CBHI regardless of the amount of payment.

### Results

A total of 604 study participants responded for the interviews, making the response rate 98.2%. All in all, 492 (81.5%) of the study participant households were willing to join the community-based health insurance scheme.

The mean age of respondents and mean family size of households were 45.86 (SD ± 12.2) and 5.06 (SD ± 1.76) respectively (Table 1).

**Table 1:** Socio-demographic and socio-economic characteristics of respondents, East Gojjam zone, 2015

Variables	Frequency	%	95% CI
*District*			
Aneded	143	23.7	20.4, 27.2
Debreliyas	176	29.1	25.6, 32.9
Goncha	101	16.7	13.9, 19.9
Bibugn	184	30.5	26.9, 34.3
*Residence*			
Urban	81	13.4	10.9, 16.4
Rural	523	86.6	83.6, 89.1
*Gender of household head*			
Male	537	88.9	86.1, 91.2
Female	67	11.1	8.8, 13.9
*Educational status of household head*			
No schooling	242	40.1	36.2, 44.0
Can read and write	232	38.4	34.6, 42.4
Primary school completed	130	21.5	18.4, 24.9
*Type of dwelling*			
Thatched	37	6.1	4.4, 8.3
Corrugated	565	93.5	91.3, 95.3
Cement	2	0.3	0.08, 1.3
*Age of household heads*			
≤ 31	67	11.1	8.8, 13.9
31–45	261	43.2	39.3, 47.2
46–60	203	33.6	29.9, 37.5
≥ 61	73	12.1	9.7, 14.9
*Household size*			
≤ 5	364	60.2	56.3, 64.1
> 5	240	39.7	35.9, 43.7
*Family member illness in the last 4 weeks*			
Yes	311	51.5	47.5, 55.5
No	293	48.5	44.5, 52.5
Mean monthly income	2142.3 ETB		1889.3, 2295.3

*ETB* Ethiopian Birr

#### Factors associated to willingness to join CBHI scheme

Bivariable and multi-variable analysis of logit model were carried out to identify factors associated with willingness to take up CBHI scheme. Variables with p-value of less than 0.2 in bivariable analysis were entered to multi-variable analyzes.

Household heads that can read and write were more likely to join the scheme compared to those who cannot read and write (AOR = 1.78; 95% CI 1.068, 2.97). Households which had experience of borrowing for medical expenses within the last 12 months prior to the study were 2.7 times more likely to join CBHI scheme than those who didn’t have borrowed (AOR = 2.65; 95% CI 1.03, 6.83). Female headed households were 2.7 times more likely to take up the scheme compare to male headed households (AOR = 2.74; 95% CI 1.18, 6.37).

In the other hand, households which own more live stocks were more likely to take up the scheme (AOR = 1.2; 95% CI 1.07, 1.34) (Table 2).

**Table 2:** Multiple logistic regression result showing the association between socio-demographic and other variables with willingness to join CBHI

Variables	AOR	P-value	95% CI	
*Educational status of household head*				
Can read and write	1.78	0.027	1.07	2.97
Cannot read and write	1.00			
*Experience of borrowing for medical expenses*				
Yes	2.66	0.042	1.03	6.83
No	1.00			
*Sex of household head*				
Male	0.36	0.02	0.16	0.84
Female	1.00			
*Residence*				
Urban	1.48	0.362	0.63	3.46
Rural	1.00			
*Experience of illness in the family within past 4 weeks*				
Yes	1.44	0.15	0.87	2.37
No	1.00			
*Age of household head*	0.98	0.149	0.96	1.01
*Family size*	1.13	0.112	0.97	1.32
*Household land size in Hectare*	0.81	0.241	0.58	1.14
*Household animal asset (in TLU)*	1.20	0.001	1.07	1.34

### Discussions

In this study 500 (85.03%) households were willing to join the CBHI scheme; this finding is higher than findings form Debub Bench District, Southwest Ethiopia [11] and Fogera District, North West Ethiopia [12] which were 77.8% and 80% in 2013 respectively. The deference might be due to time gap and level of awareness as mobilization of the scheme is being improved time to time through different Medias.

Female headed households were more likely to join CBHI scheme than male headed households; this finding is in line with findings from studies conducted in Ghana, Mali and Senegal [13], which revealed that female-headed households were more likely to enroll in CBHI schemes. This might be due to the fact that in Ethiopia most of the female headed households are managed with women without husband and are exposed for economic problems. In this case they might wish to cope with health problems joining the community based health insurance with fair payment.

But this is in opposite to a finding from Fogera district Northwest Ethiopia [12]. This difference might be related to the study area difference and the proportion of the female headed households involved in the study, as higher proportion of female household heads were involved in this study.

Credit for medical expenses is also another predictor factor for willingness to join the scheme, this finding is supported by another study in Edo state Nigeria, which showed households which had experience of borrowing for medical expenses were more likely to join CBHI scheme [14]. This might be due to the fact that households that had experience of borrowing for medical expense can recognize the economic catastrophe of out of pocket charges. So they may intend to cover such catastrophes through regular payments joining the CBHI.

Educational status of household heads positively associated with willingness to join the CBHI scheme; this finding is in line with a finding from Debub Bench District, Southwest [11] Ethiopia which showed that participants who can read and write were more likely to join CBHI scheme than those who were categorized as no education. It is also in line with a finding from Jimma, that showed individuals in tertiary educational level were more likely to be willing to join Idir based health insurance scheme as compared to illiterates [15]. Study conducted in West African countries (Gahanna, Mali, Senegal and Nigeria) also supported this finding [13, 16]. This positive relationship between education level and willingness to join CBHI can be related to the fact that as education status increased the health seeking behavior also increased. Then individuals tend to secure their family’s health status through risk spreading paying for it in advance. In addition, those individuals with better educational level could have better understanding of the concepts and principles of community based health insurance, which can ease their decision to join the scheme.

### Conclusions and recommendation

The findings of this study showed that the proportion of willingness to join the CHHI was higher than the findings in different parts of the country and it is encouraging for planned strategy of expanding the scheme throughout the country.

Educational status of household head, experience of borrowing for medical expenses, sex of household head, household animal asset as measured by tropical livestock unit were factors found to be associated with willingness to take up CBHI scheme.

## Limitations

This study claimed to generalize for relatively large area at zonal level collecting data only from four districts. The analysis is based only on fixed effects model despite covering large area. Even if the samples were selected randomly, there might be some heterogeneity among the different districts.

## Abbreviations

AOR: adjusted hazard ratio
CBHI: community based health insurance
SD: standard deviation
SHI: social health insurance
TLU: tropical livestock unit
